# Integrating additional factors into the TNM staging for cutaneous melanoma by machine learning

**DOI:** 10.1371/journal.pone.0257949

**Published:** 2021-09-30

**Authors:** Charles Q. Yang, Huan Wang, Zhenqiu Liu, Matthew T. Hueman, Aadya Bhaskaran, Donald E. Henson, Li Sheng, Dechang Chen

**Affiliations:** 1 Department of Surgery, Walter Reed National Military Medical Center, Bethesda, MD, United States of America; 2 Department of Biostatistics, The George Washington University, Washington, DC, United States of America; 3 Department of Public Health Sciences, Penn State Cancer Institute, Hershey, PA, United States of America; 4 Department of Surgical Oncology, John P. Murtha Cancer Center, Walter Reed National Military Medical Center, Bethesda, MD, United States of America; 5 Department of Quantitative Theory and Methods, Emory University, Atlanta, GA, United States of America; 6 Deceased, was with The Department of Preventive Medicine & Biostatistics, F. Edward Hébert School of Medicine, Uniformed Services University of the Health Sciences, Bethesda, MD, United States of America; 7 Department of Mathematics, Drexel University, Philadelphia, PA, United States of America; 8 Department of Preventive Medicine & Biostatistics, F. Edward Hébert School of Medicine, Uniformed Services University of the Health Sciences, Bethesda, MD, United States of America; Fondazione IRCCS Istituto Nazionale dei Tumori, ITALY

## Abstract

**Background:**

Integrating additional factors into the TNM staging system is needed for more accurate risk classification and survival prediction for patients with cutaneous melanoma. In the present study, we introduce machine learning as a novel tool that incorporates additional prognostic factors to improve the current TNM staging system.

**Methods and findings:**

Cancer-specific survival data for cutaneous melanoma with at least a 5 years follow-up were extracted from the Surveillance, Epidemiology, and End Results Program of the National Cancer Institute and split into the training set (40,781 cases) and validation set (5,390 cases). Five factors were studied: the primary tumor (T), regional lymph nodes (N), distant metastasis (M), age (A), and sex (S). The Ensemble Algorithm for Clustering Cancer Data (EACCD) was applied to the training set to generate prognostic groups. Utilizing only T, N, and M, a basic prognostic system was built where patients were stratified into 10 prognostic groups with well-separated survival curves, similar to 10 AJCC stages. These 10 groups had a significantly higher accuracy in survival prediction than 10 stages (C-index = 0.7682 vs 0.7643; increase in C-index = 0.0039, 95% CI = (0.0032, 0.0047); p-value = 7.2×10^−23^). Nevertheless, a positive association remained between the EACCD grouping and the AJCC staging (Spearman’s rank correlation coefficient = 0.8316; p-value = 4.5×10^−13^). With additional information from A and S, a more advanced prognostic system was established using the training data that stratified patients into 10 groups and further improved the prediction accuracy (C-index = 0.7865 vs 0.7643; increase in C-index = 0.0222, 95% CI = (0.0191, 0.0254); p-value = 8.8×10^−43^). Both internal validation using the training set and temporal validation using the validation set showed good stratification and a high predictive accuracy of the prognostic systems.

**Conclusions:**

The EACCD allows additional factors to be integrated into the TNM to create a prognostic system that improves patient stratification and survival prediction for cutaneous melanoma. This integration separates favorable from unfavorable clinical outcomes for patients and improves both cohort selection for clinical trials and treatment management.

## Introduction

Cutaneous melanoma is responsible for the vast majority of skin cancer-related deaths in the US and accounts for 7 percent of new cancer cases in men and 4 percent of new cancer cases in women [1]. There are multiple prognostic factors for cutaneous melanoma that correlate with survival. These include lesion thickness, lymph node involvement, metastasis, ulceration, age, sex, anatomic location, mitotic rate, satellite/in-transit lesions, and serum lactic dehydrogenase level, etc [2]. Among these, the primary tumor feature (thickness and ulceration), nodal involvement, and distant metastasis are the three factors that constitute the AJCC staging system [3].

The initial staging system for cutaneous melanoma was developed by the American Joint Committee on Cancer (AJCC) in 1977 [4]. Through the years, the AJCC makes periodic revisions to the TNM system in an effort to reflect a better understanding of the disease. The current TNM system for melanoma classifies the disease from stage I through IV. Surgical excision is often adequate for stages I-II. Sentinel lymph node biopsy may have a role in early to intermediate cases to improve staging and identify cases who may benefit from earlier lymphadenectomy [5]. Treatment of the locally advanced or metastatic disease is complex and may require additional adjuvant treatments. Multidisciplinary tumor boards are needed to individualize treatment plans for advanced stage patients. Although melanoma has been shown historically to be a radioresistant and chemoresistant cancer, clinical trials have shown the efficacy of targeted immunotherapy, especially checkpoint blockade in metastatic cases. Additional trials are ongoing to evaluate its use in the neoadjuvant, adjuvant, and palliative settings, with or without local therapy such as surgery or radiation [6].

In the context of evolving knowledge and modern tumor registries, additional factors should be integrated to augment TNM to create prognostic systems for improved research and treatment. Cox regression modeling [7] and tree modeling [8, 9] are two approaches that could expand the AJCC staging system by integrating additional factors. Cox regression, which focuses on the optimal fitting of the data, can achieve a high accuracy in predicting survival. However, the assumptions made with Cox modeling (e.g., each factor/variable makes a linear contribution to the model) usually do not prove realistic. In addition, there is no clear rule that can guide how to use the output (e.g., the nomogram) to stratify patients into risk groups analogous to AJCC stages. As a result, studies often empirically partition patients into a small number of groups (e.g., 2 or 3 quantile groups) [10]. On the other hand, the traditional survival tree method, partitioning the space of values of factors into disjoint and non-overlapping regions, can be used to form prognostic groups explicitly. However, the tree models in general have a low prediction accuracy [11].

In this study, we described a machine learning approach using the Ensemble Algorithm for Clustering Cancer Data (EACCD) [12–22] to expand the AJCC staging system by integrating additional factors that contribute to the prognosis of the disease. We demonstrated the approach by building 2 prognostic systems. One system, based on primary tumor (T), regional lymph nodes (N), and distant metastasis (M), was employed to examine the current 8th AJCC staging system. The second system, based on T, N, M, age (A), and sex (S), expanded the current staging system.

## Materials and methods

### Data

Data from patients diagnosed with cutaneous melanoma during 2004 through 2013 were obtained from 18 databases of the Surveillance, Epidemiology, and End Results Program (SEER) of the National Cancer Institute [23]. The restriction on year of diagnosis was used for reasons that 1) information of some factors’ levels was not available until 2004; 2) 2013 is the latest year that ensures a minimum 5-year follow up since the current release of SEER data followed patients up to 2018. Survival time (measured in months) and SEER cause-specific death classification variable [24] were used to obtain melanoma-specific survival for analysis. In addition to restricting the years of diagnosis between 2004 and 2013, we required patients’ nodes to be pathologically evaluated (CS Lymph Nodes Eval = 2,3,6,8 [25]). This study investigated 5 factors: T, N, M, A, and S. The datasets used for analysis were generated as follows.

**Fig 1:**
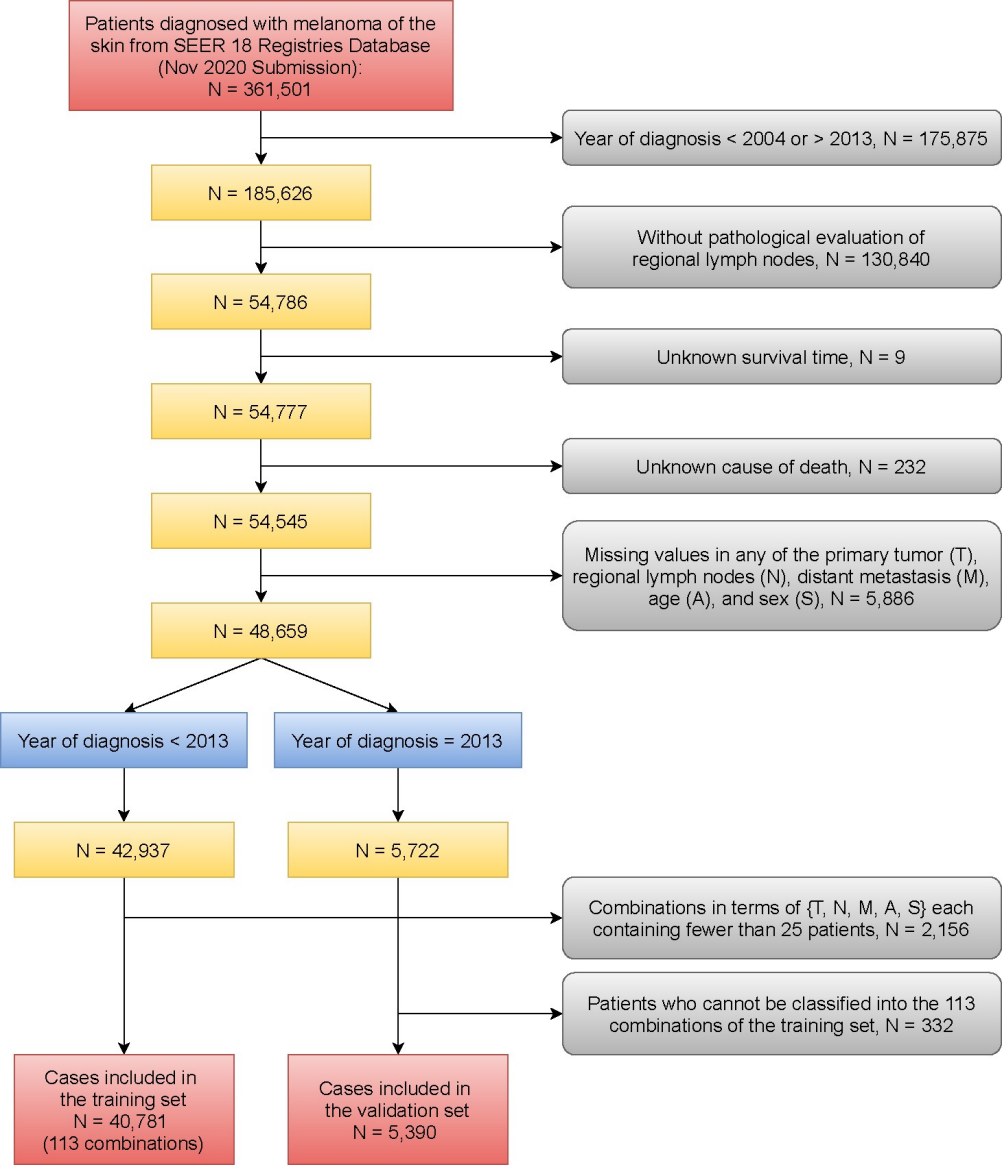
Flow diagram for selecting patients with skin melanoma.

Survival time (in months), SEER cause-specific death classification variable, T, N, M, A, and S were collected for the studied patients. The levels of T (9 levels: T0, T1a, T1b, T2a, T2b, T3a, T3b, T4a, and T4b), N (7 levels: N0, N1a, N1b, N2a, N2b, N2c, and N3), M (2 levels: M0 and M1), A (2 levels: A0, A1), and S (2 levels: S1 and S2) are defined in S1 Table. Age 70 was used as the cut-off since an age greater than 70 years was associated with worse primary tumor characteristics and higher mortality compared with younger patients [26]. A combination is defined as a subset of the data corresponding to one level of each factor and is denoted by levels of factors (e.g., T1N0M0A0S1 represents a subset of patients with T = T1, N = N0, M = M0, A = A0, S = S1). We retained patients without missing values in any of the five factors (i.e., T, N, M, A, and S) so that their combinations can be formed. Then patients were assigned to the training set if they were diagnosed with melanoma before 2013, and to the validation set if diagnosed in 2013. The validation set obtained this way was used in temporal validation to examine the reproducibility of the prognostic system built on the training set [27]. For more accurate estimation in the development of prognostic systems, we removed all rare combinations each containing less than 25 patients in the training set. The final training set used for analysis includes 113 combinations giving a total number of 40,781 cases. For the validation set, we excluded patients who could not be classified into any of the 113 combinations of the training set. The final validation set contains 5,390 patients. See Fig 1 and Table 1 for details on data. Additional information on datasets is contained in S1 Data.

**Table 1:** Clinical and demographic characteristics of the data.

	Training Set		Validation Set	
	N	%	N	%
**Primary Tumor**				
T0	68	0.2	14	0.3
T1a	8,266	20.3	984	18.3
T1b	6,929	17.0	868	16.1
T2a	10,882	26.7	1,458	27.1
T2b	2,351	5.8	358	6.6
T3a	4,456	10.9	586	10.9
T3b	3,097	7.6	441	8.2
T4a	1,877	4.6	267	5.0
T4b	2,855	7.0	414	7.7
**Regional Lymph Nodes**				
N0	34,995	85.8	4,647	86.2
N1a	3,240	7.9	404	7.5
N1b	302	0.7	41	0.8
N2a	861	2.1	113	2.1
N2b	238	0.6	46	0.9
N2c	154	0.4	20	0.4
N3	991	2.4	119	2.2
**Distant Metastasis**				
M0	40,739	99.9	5,389	100.0
M1	42	0.1	1	0.0
**Age**				
A0	29,738	72.9	3,738	69.4
A1	11,043	27.1	1,652	30.6
**Sex**				
S1	24,861	61.0	3,384	62.8
S2	15,920	39.0	2,006	37.2

### EACCD

The EACCD is a machine learning algorithm that can be used to cluster patients into n* prognostic groups so that i) patients from the same group have a similar survival experience and patients from two distinct groups have different survival experiences; and ii) n* is as small as possible while n* groups can achieve the maximum possible accuracy in survival prediction. In this study, we will use an expanded version of the algorithm that consists of 4 steps: 1) defining initial dissimilarities between survival functions of any two combinations; 2) obtaining learned dissimilarities by using initial dissimilarities and an ensemble learning method; 3) applying hierarchical clustering to cluster combinations by the learned dissimilarities; and 4) generating n* prognostic groups by exploiting the C-index. Different approaches are available for each step. In this paper, the initial dissimilarity between two combinations in step 1 was defined by Mann-Whitney based effect size |*P*(*T*_1_>*T*_2_)−0.5|, where *T*_1_ and *T*_2_ are the failure times of two underlying populations corresponding to the two combinations (see below). In step 2, the ensemble learning method was based on the two-phase Partitioning Around Medoids algorithm [28]. The minimax linkage method [29] was chosen for hierarchical clustering in step 3. The output of this step is a tree-structured dendrogram, which represents the relationship among survival of patients in different combinations. In step 4, a C-index curve was first produced using the dendrogram in step 3 and then the optimal number n* of prognostic groups was found using the “knee” point of the C-index curve and then the n* prognostic groups were generated by cutting the dendrogram [16, 18, 20].

### Mann-Whitney based effect size

The Mann-Whitney parameter arises from the widely used Mann-Whitney test [30] that examines whether one of two random variables is stochastically larger than the other. Let *T*_1_ and *T*_2_ denote the variables of survival time for patients from population 1 (with survival function *S*_1_(*t*)) and population 2 (with survival function *S*_2_(*t*)), respectively. The Mann-Whitney parameter is defined as *P*(*T*_1_>*T*_2_), the probability that a randomly chosen patient from population 1 has a longer survival time than a randomly chosen patient from population 2. The difference between the Mann-Whitney parameter and 0.5 suggests a difference between *S*_1_(*t*) and *S*_2_(*t*). Efron proposed to use $\hat{D}=-\int_{0}^{\infty }{\hat{S}}_{1}(t){d\hat{S}}_{2}(t)$ to estimate the Mann-Whitney parameter for censoring data, where ${\hat{S}}_{1}(t)$ and ${\hat{S}}_{2}(t)$ are Kaplan-Meier estimates of *S*_1_(*t*) and *S*_2_(*t*), respectively [31]. However, Efron’s estimator requires that both ${\hat{S}}_{1}(t)$ and ${\hat{S}}_{2}(t)$ drop to 0 at the maximum study time (the longest following-up time) and is rather unstable when censoring occurs due to incomplete follow up [32]. To overcome this problem, Wang [20] proposed to 1) use the exponential tails to complete survival functions; and 2) use the completed survival curves to approximate *P*(*T*_1_>*T*_2_). More specifically, *P*(*T*_1_>*T*_2_) is approximated by *D*_*e*_(*τ*_1_, *τ*_2_) = *A*+*B*+*C*, where $A=-\int_{0}^{min(\tau_{1},\tau_{2})}S_{1}(t){dS}_{2}(t);\;B=-\int_{\tau_{1}}^{\tau_{2}}e^{-\lambda_{1}t}{dS}_{2}(t)$ if *τ*_1_<*τ*_2_, 0 if $\tau_{1}=\tau_{2},\;\int_{\tau_{2}}^{\tau_{1}}S_{1}(t)\lambda_{2}e^{-\lambda_{2}t}dt$ if *τ*_1_>*τ*_2_; $C=\frac{\lambda_{2}}{\lambda_{1}+\lambda_{2}}e^{-(\lambda_{1}+\lambda_{2})max(\tau_{1},\tau_{2})}$; and *λ*_*i*_ = −*log*(*S*_*i*_(*τ*_*i*_))/*τ*_*i*_ for *i* = 1, 2. Accordingly, ${\hat{D}}_{e}(\tau_{1},\tau_{2})=\hat{A}+\hat{B}+\hat{C}$, an estimate of *P*(*T*_1_>*T*_2_), can be obtained by using the Kaplan-Meier estimates and Lebesgue–Stieltjes integration. In this paper, we used $\left|{\hat{D}}_{e}(\tau_{1},\tau_{2})-0.5\right|$ to compute the initial dissimilarity in survival between two combinations, with *τ*_1_ and *τ*_2_ set to be the maximum possible time by which the Kaplan-Meier estimates of the survival of all combinations can be calculated.

### Prognostic systems

Survival curves using the Kaplan-Meier estimates [33] were plotted for the n* prognostic groups from EACCD. These curves allow visually evaluating survival differences among the groups. The final prognostic system includes the dendrogram, group assignment, C-index, and survival curves for the prognostic groups.

### Validation approach

The prognostic system was evaluated through both internal and temporal validation [27]. Internal validation, conducted on the training set, was mainly used to evaluate the validity of the system. Stratification and the predictive accuracy, two key characteristics of the system, were evaluated with internal validation. Stratification was examined by inspecting the survival curves of prognostic groups and comparing them using the univariate Cox proportional hazards regression model and the logrank test. The predictive accuracy was examined by computing the C-index of the prognostic system. The temporal validation, conducted on the validation set, was used to assess the system’s reproducibility, i.e., both stratification and the predictive accuracy obtained using the training set were also valid for patients in the validation set.

### Software

All statistical analyses were done in R (R version 3.6.2) using the following libraries: survival, cluster, protoclust, factoextra, and compareC.

## Results

### Prognostic system for T, N, M

**Fig 2:**
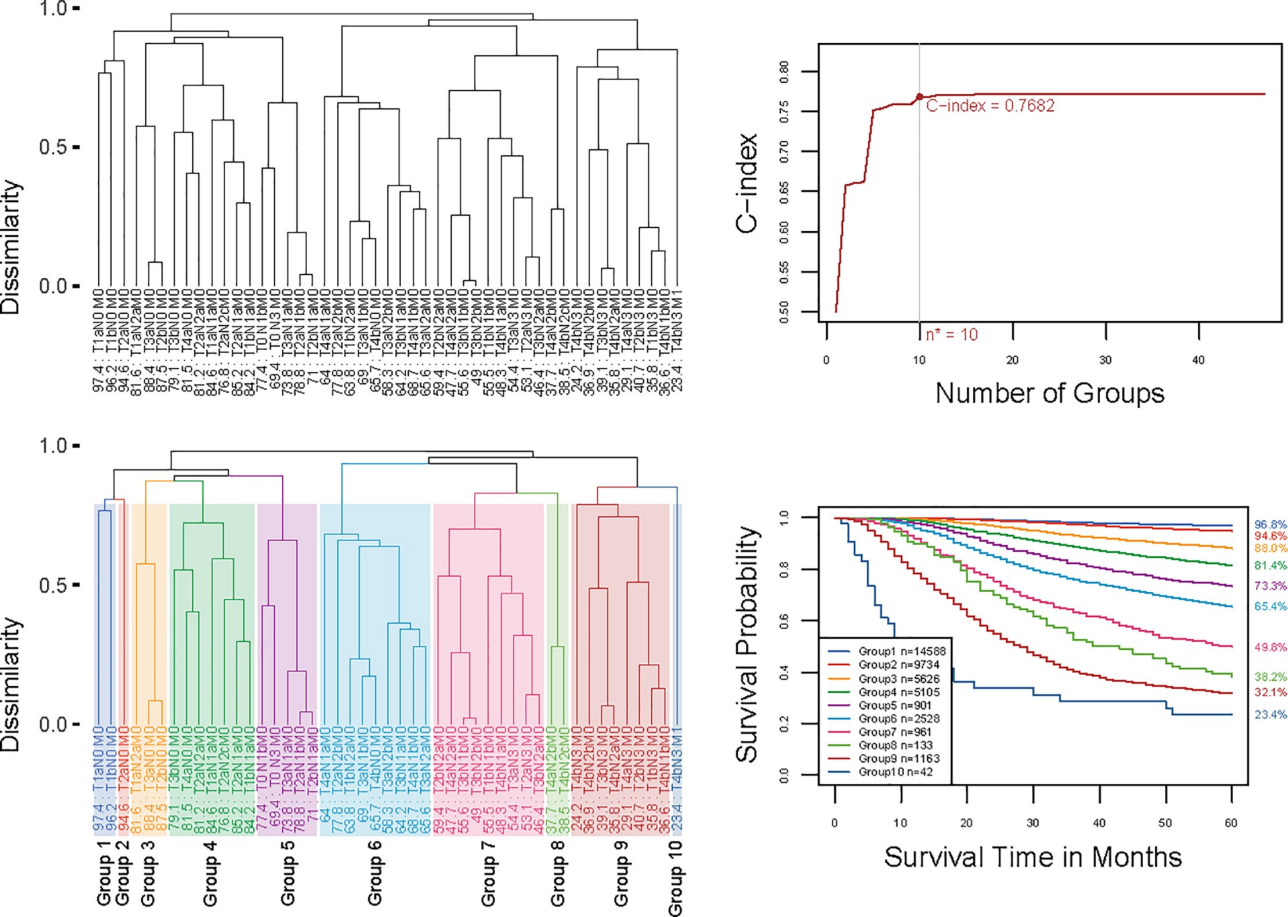
Creating EACCD prognostic groups on T, N, and M by the training set. **A** Dendrogram from running EACCD. A 5-year melanoma-specific survival rate in percentage is provided below each combination. **B** C-index curve based on the dendrogram in panel **A**. The knee point of the curve corresponds to 10 groups and a C-index value of 0.7682. **C** Cutting the dendrogram in panel **A** according to n* = 10 suggested in panel **B** creates 10 prognostic groups, shown in rectangles. Listed on the bottom of the dendrogram are group numbers. **D** Melanoma-specific survival of 10 prognostic groups in panel **C**. The 5-year melanoma-specific survival rates for 10 groups are listed on the right side of the figure.

**Fig 3:**
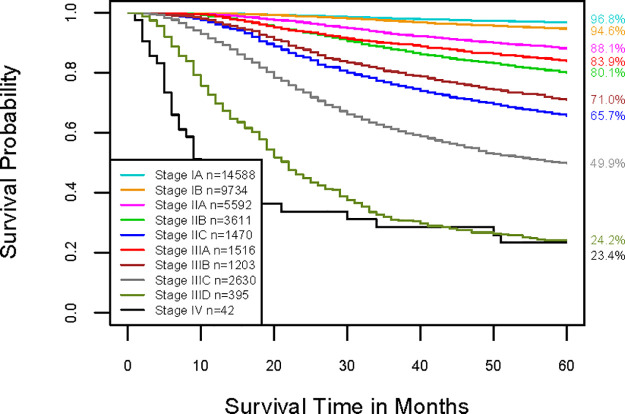
Melanoma-specific survival of AJCC stages of the training data. Stages are defined in the 5th column in S2 Table. The 5-year melanoma-specific survival rates for 10 stages are listed on the right side of the figure.

Applying the EACCD with T, N, and M to the training set yielded the dendrogram in Fig 2A. Dissimilarity between two combinations was computed by completing survival curves of combinations with exponential tails from 149 months, i.e., *τ*_1_ = *τ*_2_ = 149. The C-index curve based on the dendrogram, shown in Fig 2B, was used to find the optimal number of prognostic groups n*. The knee point of the curve corresponds to 10 groups (C-index value of 0.7682), which suggested n* = 10. Cutting the dendrogram into n* = 10 groups is shown by rectangles in Fig 2C. Fig 2D (and S1 Fig) shows the survival curves of these 10 groups, which are well separated. For convenience, the definition for all 10 groups is restated in the 4th column of S2 Table. The resulting prognostic system for T, N, and M includes the dendrogram in Fig 2A, the groups in S2 Table (4th column), and the survival curves in Fig 2D. Note that the risk of mortality is increased as the group number increases. For a quick comparison, Fig 3 (and S2 Fig) shows the survival curves of 10 stages of the 8th AJCC staging system.

### Prognostic system for T, N, M, A, S

When T, N, M, A, and S were involved, the dissimilarity between two combinations was computed by completing survival curves of combinations with exponential tails from 115 months, i.e., *τ*_1_ = *τ*_2_ = 115.

**Fig 4:**
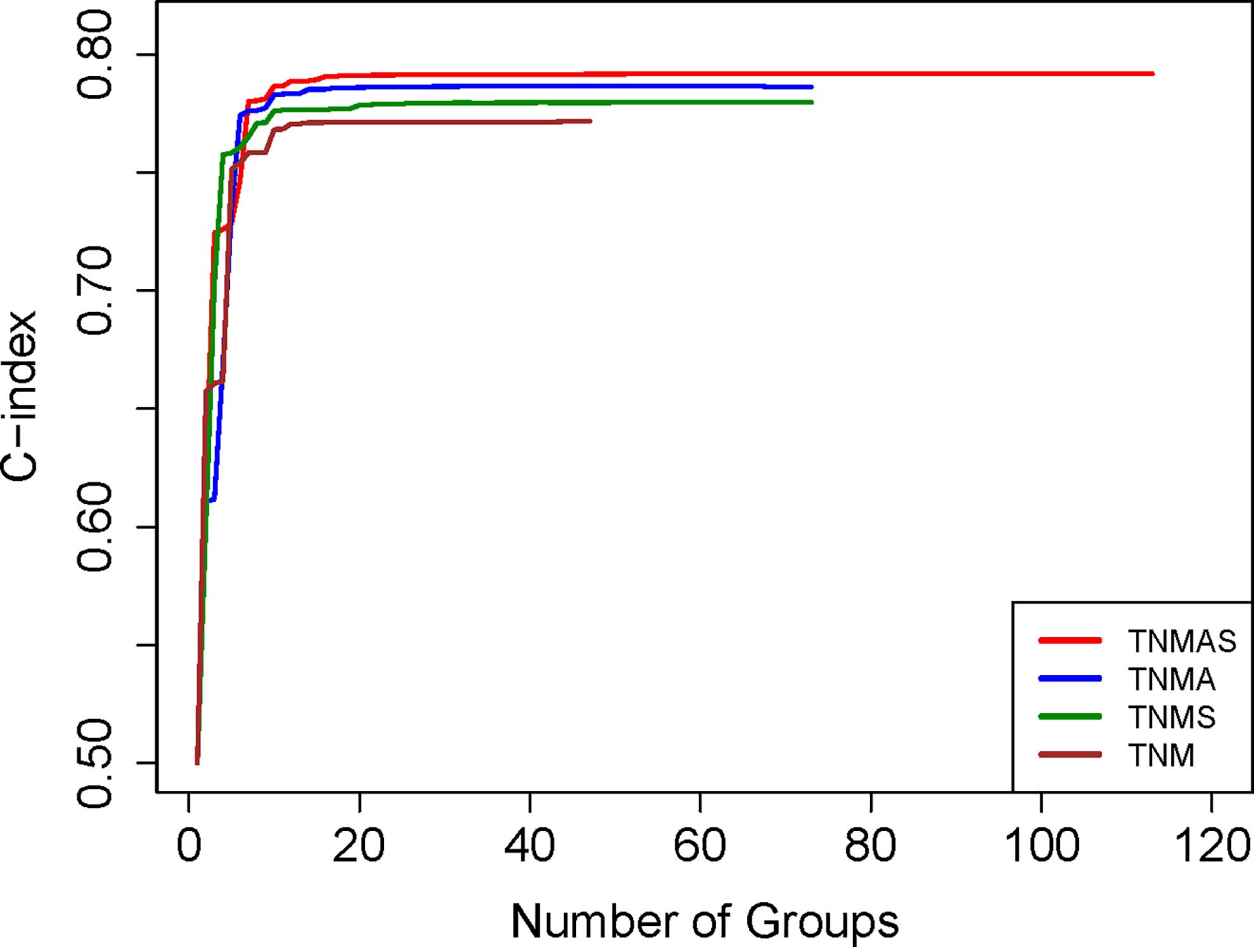
Training data C-index curves based on different factors.

Before building the EACCD prognostic system for T, N, M, A, and S by the training set, we assessed the performance in survival prediction of the models for the following three sets of factors: {T, N, M, A}, {T, N, M, S}, and {T, N, M, A, S}, as compared with the model based only on {T, N, M}. Fig 4 plots the C-index curves of the training set, each created in step 4 of EACCD, for these 4 scenarios. It is seen that the curves on {T, N, M, A} and {T, N, M, S} are higher than the curve shown on {T, N, M}. Therefore, adding A or S to {T, N, M} increases the C-index and thus improves the prediction accuracy. The curve of {T, N, M, A, S} is the highest among all 4 curves, implying that adding both A and S to {T, N, M} leads to the biggest improvement in prediction accuracy of {T, N, M}.

**Fig 5:**
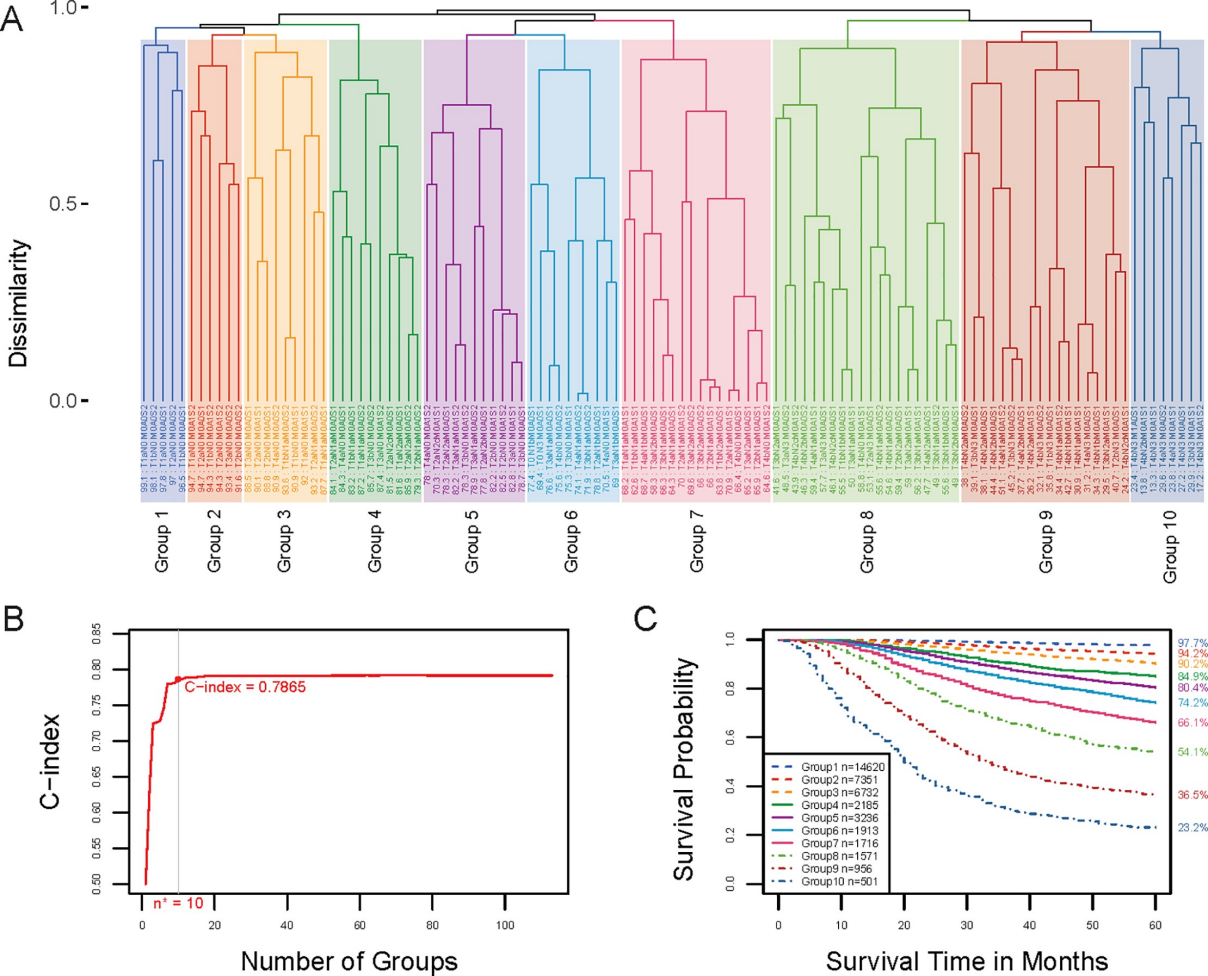
Creating the EACCD prognostic groups on T, N, M, A, and S by the training set. **A** Dendrogram and cutting the dendrogram (shown in rectangles) according to the C-index. Running the EACCD results in the tree-structured dendrogram. A 5-year melanoma-specific survival rate in percentage is provided below each combination. Cutting the dendrogram according to n* = 10 in panel **B** creates 10 prognostic groups, shown in rectangles. Listed on the bottom of the dendrogram are group numbers. **B** C-index curve based on the dendrogram in panel **A**. The knee point of the curve corresponds to 10 groups and a C-index value of 0.7865. **C** Melanoma-specific survival of 10 prognostic groups in panel **A**. The 5-year melanoma-specific survival rates for 10 groups are listed on the right side of the figure.

Exploring around the “knee” point of the red curve on {T, N, M, A, S} in Fig 5B reveals the optimal number of prognostic groups n* = 10 with a corresponding C-index of 0.7865. Fig 5A shows the dendrogram and its cutting (shown in rectangles) according to n* = 10 groups. A detailed definition for all 10 groups is listed in S3 Table. Fig 5C shows the survival curves for the 10 prognostic groups. The dendrogram in Fig 5A, the groups in S3 Table, and the survival curves in Fig 5C (and S3 Fig) present a prognostic system for T, N, M, A, and S.

### Validation of the EACCD systems

The EACCD systems in {T, N, M} and {T, N, M, A, S} built above can be validated internally using the training data and temporally using the validation data. Below we present validation results for the EACCD system based on {T, N, M, A, S}. The same procedure can be followed for the EACCD system on{T, N, M}.

The internal validation evaluates the stratification and predictive accuracy of the EACCD system using only the training set. Fig 5 indicates that the survival curves of the prognostic groups are well separated. This good separation can be verified from the comparison between survival curves of adjacent groups, shown in Table 2. This table reveals that the hazard ratios between adjacent groups are uniformly significant and are mostly between 1.3 and 1.6., and that the logrank test results between adjacent groups are also uniformly significant. The previous section showed that the C-index value of the EACCD system based on {T, N, M, A, S} has a C-index value close to 0.8, which implies a good predictive accuracy of the system [27]. Therefore, the EACCD system on {T, N, M, A, S} well stratifies and predicts the patients from the training set.

**Table 2:** Output of the Cox proportional hazards regression model and the logrank test for EACCD grouping on the basis of T, N, M, A, and S on the training set.

	Training Set			Validation Set		
Groups Compared	Hazard Ratio (95% CI)	P-value for Hazard Ratio	P-value for Logrank Test	Hazard Ratio (95% CI)	P-value for Hazard Ratio	P-value for Logrank Test
2 vs 1	2.52 (2.26,2.81)	6.9×10^−62^	3.0×10^−66^	2.89 (1.87,4.47)	1.6×10^−6^	5.0×10^−7^
3 vs 2	1.60 (1.45,1.76)	8.5×10^−21^	3.8×10^−21^	1.66 (1.17,2.36)	0.0046	0.0042
4 vs 3	1.53 (1.37,1.71)	6.4×10^−14^	4.2×10^−14^	1.38 (0.92,2.06)	0.12	0.12
5 vs 4	1.30 (1.16,1.46)	6.4×10^−6^	6.0×10^−6^	1.42 (0.95,2.13)	0.089	0.088
6 vs 5	1.29 (1.16,1.44)	3.5×10^−6^	3.3×10^−6^	1.48 (1.07, 2.05)	0.019	0.018
7 vs 6	1.37 (1.22,1.53)	4.3×10^−8^	3.8×10^−8^	1.10 (0.77,1.58)	0.60	0.60
8 vs 7	1.45 (1.31,1.61)	1.8×10^−12^	1.4×10^−12^	2.01 (1.43,2.83)	5.9×10^−5^	4.2×10^−5^
9 vs 8	1.66 (1.50,1.85)	8.6×10^−21^	3.6×10^−21^	1.35 (0.98,1.85)	0.062	0.062
10 vs 9	1.59 (1.40,1.82)	1.9×10^−12^	1.3×10^−12^	1.44 (0.96,2.17)	0.080	0.079

The p-value for the hazard ratio is from the Wald test testing the null hypothesis that the hazard ratio equals 1. The p-value for the logrank test is from the logrank test testing the null hypothesis that two survival curves are equal.

**Fig 6:**
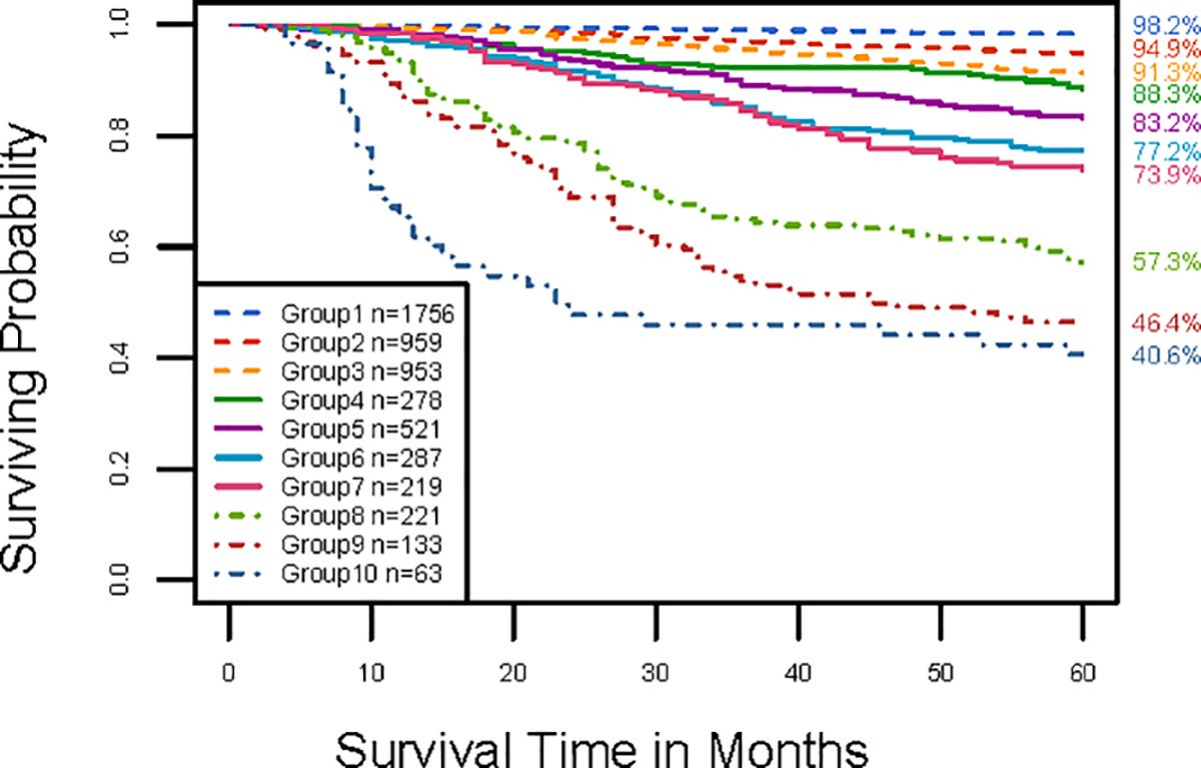
Melanoma-specific survival of the 10 prognostic groups from the validation set. These groups were obtained by applying to the validation set the assignment rule of the EACCD system based on the training set. The 5-year melanoma-specific survival rates for 10 groups are listed on the right side of the figure.

The external validation examines the stratification and predictive accuracy of the EACCD system on the validation set. Fig 6 (and S4 Fig) shows the survival curves of the 10 prognostic groups on the validation set. Although due to the small sample size these curves do not provide accurate estimates of the survival rates, they preserve the pattern from the training set (Fig 5). This is further confirmed by Table 2, which shows separation between groups, though some results are less significant due to small sample sizes. Thus a good stratification occurs in the validation set. (The large p-value between groups 6 and 7 is mainly due to their partially overlapping survival curves in early years (Fig 6 and S4 Fig).) Calculation shows that the EACCD system has a C-index value of 0.7941 on the validation set, which is high. Therefore the system has a high predictive accuracy for the patients in the validation set.

The above internal and external validation shows that the EACCD system on {T, N, M, A, S} has good stratification and a high predictive accuracy for patients used in the development and patients in the future. Applying a similar validation procedure to the EACCD system on {T, N, M} led to the same conclusion for the system in three variables.

## Discussion

### Comparison with AJCC

With the training data, the EACCD prognostic system on {T, N, M} can be compared with the 8th ed. AJCC staging system in terms of both prediction and stratification.

The 8th edition of AJCC divides the training set into 10 stages (The 5th column of S2 Table and Fig 3). Calculation shows that the AJCC staging system has a C-index of 0.7643. The p-value of the non-parametric C-index based test [34] for testing the difference (0.0039, 95% CI = (0.0032, 0.0047)) between the prediction accuracy of the above EACCD prognostic system (10 groups, C-index = 0.7682) and the AJCC staging system TNM (10 stages, C-index = 0.7643) is 7.2×10^−23^, showing that the former has a significantly higher survival prediction accuracy than the latter.

Table 3 presents the distribution of patients for each of the 10 AJCC stages over the 10 EACCD groups. Roughly, it is seen that a higher stage in AJCC corresponds to a higher-risk group in EACCD, and vice versa. Indeed the assignment to ordered stages (from the smallest to the biggest risk) and the assignment to ordered prognostic groups have a large Spearman’s rank correlation coefficient [35] of 0.8316 with a p-value of 4.5×10^−13^. Therefore, there is a strong positive association between AJCC staging and EACCD grouping.

**Table 3:** Contingency table between EACCD grouping and AJCC staging on the basis of T, N, M on the training set.

_AJCC_\^EACCD^	1	2	3	4	5	6	7	8	9	10	Total
**IA**	14,588	0	0	0	0	0	0	0	0	0	14,588
**IB**	0	9,734	0	0	0	0	0	0	0	0	9,734
**IIA**	0	0	5,592	0	0	0	0	0	0	0	5,592
**IIB**	0	0	0	3,611	0	0	0	0	0	0	3,611
**IIC**	0	0	0	0	0	1,470	0	0	0	0	1,470
**IIIA**	0	0	34	1,439	0	43	0	0	0	0	1,516
**IIIB**	0	0	0	0	864	274	65	0	0	0	1,203
**IIIC**	0	0	0	55	37	741	896	133	768	0	2,630
**IIID**	0	0	0	0	0	0	0	0	395	0	395
**IV**	0	0	0	0	0	0	0	0	0	42	42
**Total**	14,588	9,734	5,626	5,105	901	2,528	961	133	1,163	42	40,781

However, survival curves of different AJCC stages often overlap, which can cause confusion in treatment planning. For instance, stages IIID and IV have overlapping survival curves (Fig 3 and S2 Fig). In addition, due to heterogeneity, higher stage groups can have higher survival rates, which is counterintuitive. For example, stage IIIA has a more favorable survival curve than stage IIB, and stage IIIB has a more favorable survival curve than stage IIC. These drawbacks were also reported by Abdel-Rahman et al. [36], though they applied a different data management process to the SEER data. We must emphasize that the EACCD prognostic groups rarely have these problems—the survival curves are well separated and the index of prognostic groups preserves the risk order. Table 3 details how this happens.

In fact, Table 3 shows whether cases of an AJCC stage should be divided according to prognostic groups included in the EACCD. Horizontally, we can examine all stages line by line. First, we observe that no splitting for stage IA and all 14,588 cases are assigned to group 1. Then we observe all cases included in stage IB are assigned to prognostic group 2. We continue until reaching the last line where stage IV equals prognostic group 10. The key observation is that each of stages IIIA, IIIB, and IIIC is split into cases of multiple prognostic groups. For instance, cases of stage IIIC are decomposed into those in prognostic groups 4–9. We note that based on its design, EACCD grouping represents an assignment of cases so that cases within a group are more homogeneous in survival and cases from different groups differ in survival. Therefore, the above observation may indicate that each of stages IIIA, IIIB, and IIIC contains more heterogeneous cases than other stages. This is consistent with the AJCC definition of stages IIIA, IIIB, and IIIC [3].

In summary, in predicting survival, the EACCD prognostic system on {T, N, M} has a significantly higher accuracy than the AJCC staging system TNM. In stratifying patients, the EACCD grouping produces well-separated survival curves while AJCC staging does not, but EACCD grouping and AJCC staging are strongly positively associated.

### Effect of factor levels on survival

The EACCD prognostic system on {T, N, M, A, S} represents an expansion to TNM. Compared with the EACCD system on {T, N, M}, this expanded system further improved the prediction accuracy of the AJCC staging system (C-index = 0.7865 vs 0.7643; increase in C-index = 0.0222, 95% CI = 0.0191, 0.0254); p-value = 8.8×10^−43^).

**Fig 7:**
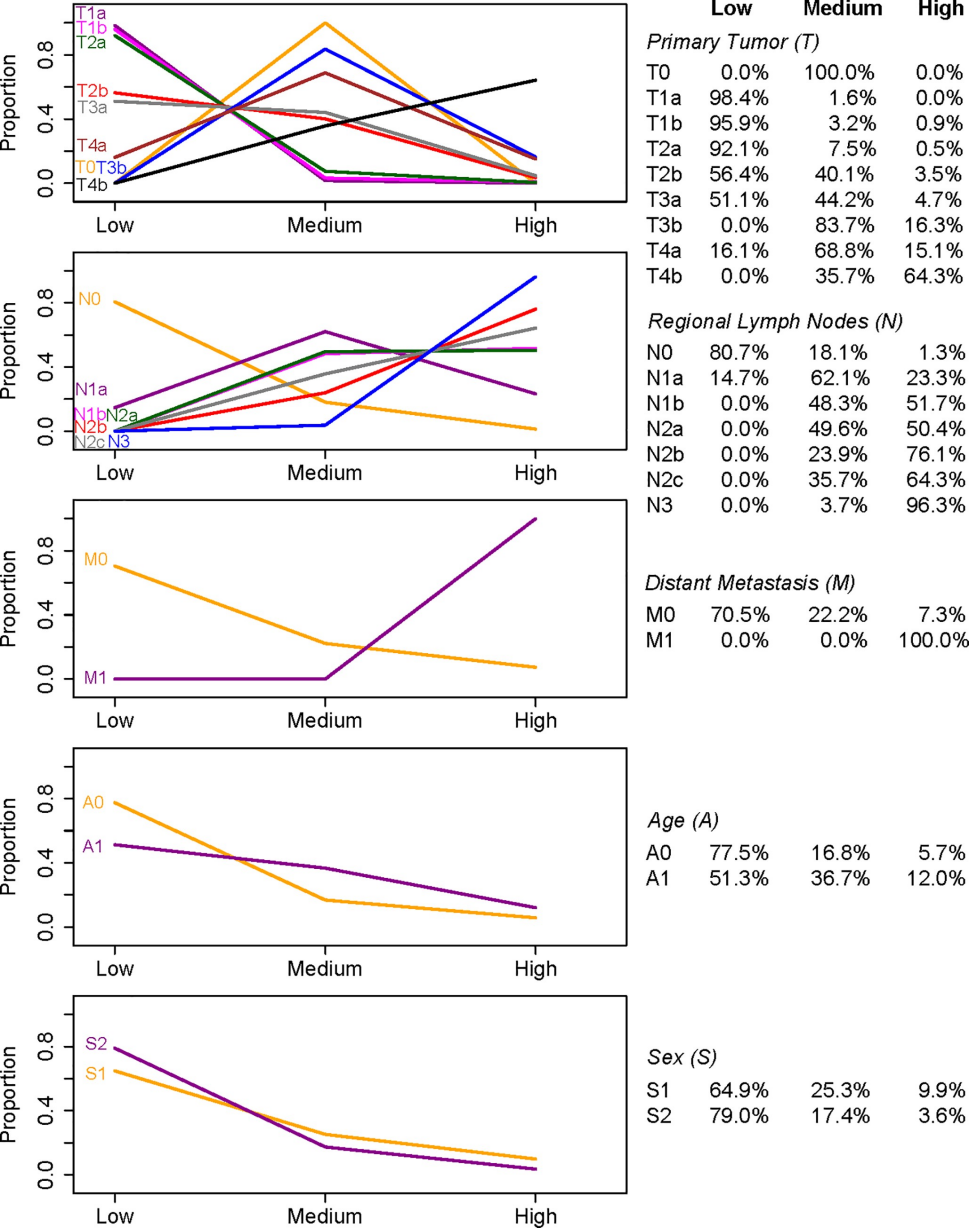
Distributions of patients over risk categories on the training set. In each panel, one factor is concerned, and for each level of the factor, the distribution of patients (3 proportions at 3 risk categories) is presented in two ways: plot on the left and tabulation on the right.

Below we applied this expanded system to examine the effect of levels of individual factors on survival. To simplify the analysis, we consider the following three risk categories by combining 10 prognostic groups from the system: low risk (groups 1–3), medium risk (groups 4–7), and high risk (groups 8–10). Fig 7 shows how patients associated with a factor level are distributed across these three risk categories.

The 1^st^ panel of Fig 7 shows the distribution of patients associated with different T levels. T1a, T1b, and T2a curves peak at low risk with a near 100% proportion, indicating that patients associated with T1a, T1b, and T2a are almost exclusively distributed in groups with low risk. The T2b and T3a curves also peak at low risk. Patients with these two levels are more likely to appear in low-risk groups, but there is a reasonably big proportion (about 40%) of them classified into middle-risk groups. Patients with T0, T3b, and T4a are mostly distributed in middle-risk groups. We note that T0 is not a highly optimistic level as what we would typically imagine. The reason is that patients with T0 are always associated with N levels higher than N0. The indication of a medium risk is also reflected in the AJCC staging system where T0 patients are assigned to either stage IIIB or IIIC. T4b is the level with the worst prognosis and patients with T4b are mostly distributed in high-risk groups. The above indicates an increased severity of the disease in terms of the order {T1a, T1b, T2a}, {T2b, T3a}, {T0, T3b, T4a}, T4b.

The 2^nd^ panel shows that: 1) N0 patients are most likely to have a low risk; 2) N1a patients are more likely to assume a medium risk; 3) N1b and N2a patients are most likely at either a medium or high risk; and 4) N2b, N2c, and N3 patients are most likely to have a high risk. These indicate the increased severity of the disease in terms of the order N0, N1a, {N1b, N2a}, {N2b, N2c, N3}.

The 3^rd^ panel reveals that M0 patients have a decreasing distribution from low to high-risk groups, whereas the entire M1 patients are distributed in the high-risk group only. This clearly suggests that patients with M1 have a poor prognosis.

The 4^th^ and 5^th^ panels clearly show that A0 and S2 have a better prognosis than A1 and S1, respectively.

The above analysis shows how a factor level is associated with risk. Although these findings are known from the literature, this is the first time these factor levels are integrated together and described explicitly in ordered risk groups of the prognostic system TNMAS created in this paper.

### Relationship to stratification and prediction

It is seen that an EACCD prognostic system is derived by partitioning, according to survival, patients into a minimum number of groups that yields approximately the maximum value of the predictive accuracy, i.e., C-index. A prognostic system is neither a model only targeting at the “best” stratification nor a model only aiming at the “highest” predictive accuracy. A prognostic system is a model that shows a tradeoff between stratification and prediction. It is this balance that possesses unique utilizations in clinical settings such as staging patients, offering treatment, and designing trials, etc.

### Limitations of analyses

Melanoma-specific survival data were used in this study. Although the SEER cause-specific death classification is determined according to various features (e.g., tumor sequence, site of the original cancer diagnosis, and comorbidities), death certificate errors could introduce a bias to the estimation of cause-specific survival. Another limitation is that the EACCD requires a relatively large data set in order to obtain robust estimates of survival. This report includes combinations with a minimum of 25 cases, which may exclude some “rare” but interesting combinations. The impact of this requirement on the size of combinations will be minimized as more data become available. In addition, the absence of N1c subcategory in our study could affect, to some extent, the comparison between our system and the AJCC system (S1 Data).

## Conclusions

We used SEER data and the EACCD to build two prognostic systems to demonstrate how machine learning techniques can be used to improve prognostic assessment and risk stratification for cutaneous melanoma. The basic system, on the basis of T, N, and M, classifies patients into prognostic groups that are strongly positively correlated with the AJCC TNM stages but have a higher prediction accuracy in survival. Survival curves of the prognostic system from EACCD do not overlap and are reasonably ordered, which is in contrast to the AJCC TNM system where different stages can present overlapping survival. The second system, incorporating age and sex as additional prognostic factors, further improves prediction accuracy. The prognostic systems from EACCD serve the same role as the TNM staging system and are created through optimizing both prediction and stratification, which can impact clinical and treatment decisions.

As our understanding of melanoma and other cancers improves, more factors will come to the forefront of clinical significance. These additional factors will be very difficult to manually incorporate into conventional TNM staging systems. Our approach to staging allows incorporation of unlimited additional factors to create nuanced staging systems that will provide real-time prognosis information. When sufficient data become available, other variables/factors, such as treatment and immune checkpoint inhibitors, could be readily integrated into known systems by the EACCD to generate prognostic systems for refinements in stratification and outcome prediction that are necessary for patient care, such as monitoring large therapeutic trials.

## Supporting information

S1 Data Supplementary data. (DOCX)

S1 Fig Fig 2D with the number of patients at risk at different times. (TIF)

S2 Fig Fig 3 with the number of patients at risk at different times. (TIF)

S3 Fig Fig 5C with the number of patients at risk at different times. (TIF)

S4 Fig Fig 6 with the number of patients at risk at different times. (TIF)

S1 Table Definitions of levels of T, N, M, A, and S for SEER melanoma of the skin. Refer to AJCC Cancer Staging Manual [1] and SEER Research Data Record Description [2] for specifics of the 3rd column.
(DOCX)

S2 Table EACCD and AJCC grouping of melanoma of the skin patients according to T, N, and M. (DOCX)

S3 Table EACCD grouping of melanoma of the skin patients according to T, N, M, A, and S. (DOCX)
