# Supplementary material for: Integrating additional factors into the TNM staging for cutaneous melanoma by machine learning
Source: PLoS One. 2021 Sep 30;16(9):e0257949. doi: 10.1371/journal.pone.0257949 (PMC8483349; doi:10.1371/journal.pone.0257949)
Supplement: S1 Data — (DOCX) [file pone.0257949.s001.docx]

**S1 Data. Supplementary Data.**

The anatomic factors primary tumor (T), regional lymph nodes (N), and distant metastasis (M) are the most important prognostic factors for prognosticating melanoma of the skin [1]. We employed the latest definitions of T, N, and M in the 8th AJCC staging system for our study. However, the current release of SEER does not include categories of T, N, and M that adapt to the 8th edition. Therefore, we rebuilt the T, N, and M categories according to the 8th edition by modifying an earlier edition of T, N, and M categories recorded in SEER. To be specific, this transformation was based on the *Derived AJCC-6 T, N, M* variables [2] that match T, N, M categories for the 6th AJCC cancer staging systems. Note that the *Derived AJCC-6 T, N, M* variables were initially included in SEER during 2004.

There was no significant change in the definition for T among the 6^th^ [3], 7th [4], and 8^th^ [1] AJCC cancer staging systems except for T1. Besides tumor thickness (or Breslow depth) and ulceration, the Clark level was used to determine the subcategories for T1 in the 6th AJCC staging system while mitotic rates took its place in the 7th AJCC staging system. The 8th AJCC staging system kept only thickness and ulceration status as the classification criteria for T1 tumors. The information of thickness and ulceration status is available in SEER [5, 6], which can be used to reclassify the T1 category of *Derived AJCC-6 T* according to the 8th edition of T definition.

Pathological evaluation of N is required for pathological staging of melanoma. Compared to the definition of N in the 6th staging manual, the empirically defined "microscopic" and "macroscopic" descriptors were redefined as “clinically occult” and “clinically detected” regional node disease in the 8th edition, respectively. Additional noteworthy changes to the definition of N categories are described as follows. One change is that the N1 category in the 8th edition included an additional subcategory N1c. Unfortunately, classifying N1c requires the information of the presence of in-transit, satellite, and/or microsatellite metastases. Such information is not available in SEER data, and as a result, we were unable to use N1c in our study. Another change is that the 8th staging manual further divides N3 categories into N3a, N3b, and N3c. There is no difference among these three subcategories in determining the stage according to the 8th AJCC staging system except for patients diagnosed with T0 and M0, who are assigned to stage IIIC if diagnosed with N3b or N3c and have no assignment if diagnosed with N3a. In our study, we used N3 instead of its subcategories in building our system.

For the distant metastasis status, we used the main categories M0 and M1 in our system. Note that the same categories were used in the AJCC staging system 8th edition.

In addition to T, N, and M, age (A) and sex (S) were also studied in this paper since they have been considered important factors in survival prediction [7-9].

1. Amin MB, Edge S, Greene F, Byrd DR, Brookland RK, Washington, MK, et al. AJCC Cancer Staging Manual, 8th ed. Springer International Publishing. 2017.
2. SEER Research Data Record Description. Available online at: https://seer.cancer.gov/data-software/documentation/seerstat/nov2020/TextData.FileDescription.pdf (accessed 20 June 2021).
3. Greene FL, Page DL, Fleming ID, Fritz AG, Balch CM, Haller DG, et al. AJCC Cancer Staging Manual, 6th ed. New York: Springer-Verlag; 2002. <https://cancerstaging.org/references-tools/deskreferences/documents/ajcc6thedcancerstagingmanualpart2.pdf> (accessed 20 June 2021).
4. Edge S, Byrd DR, Compton CC, Fritz AG, Greene FL, Trotti A. AJCC Cancer Staging Manual, 7th ed. New York: Springer; 2010. <http://dev.cancerstaging.org/references-tools/deskreferences/Documents/AJCC%207th%20Ed%20Cancer%20Staging%20Manual.pdf> (accessed 20 June 2021).
5. CS Site-Specific Factor 1. Available online at:

<https://staging.seer.cancer.gov/cs/input/02.05.50/melanoma_skin/ssf1/?breadcrumbs=(~schema_list~),(~view_schema~,~melanoma_skin~)> (accessed 20 June 2021).

1. CS Site-Specific Factor 2. Available online at:

<https://staging.seer.cancer.gov/cs/input/02.05.50/melanoma_skin/ssf2/?breadcrumbs=(~schema_list~),(~view_schema~,~melanoma_skin~)> (accessed 20 June 2021).

1. Austin PF, Cruse CW, Lyman G, Schroer K, Glass F, Reintgen DS. Age as a prognostic factor in the malignant melanoma population. Ann Surg Oncol. 1994 Nov 1;1(6):487-94.
2. Chao C, Martin RC, Ross MI, Reintgen DS, Edwards MJ, Noyes RD, et al. Correlation between prognostic factors and increasing age in melanoma. Ann Surg Oncol. 2004 Mar 1;11(3):259-64.
3. Bartlett EK, Karakousis GC. Current staging and prognostic factors in melanoma. Surg Oncol Clin. 2015 Apr 1;24(2):215-27.
