# Supplementary figures and images for: Integrating additional factors into the TNM staging for cutaneous melanoma by machine learning
Source: PLoS One. 2021 Sep 30;16(9):e0257949. doi: 10.1371/journal.pone.0257949 (PMC8483349; doi:10.1371/journal.pone.0257949)

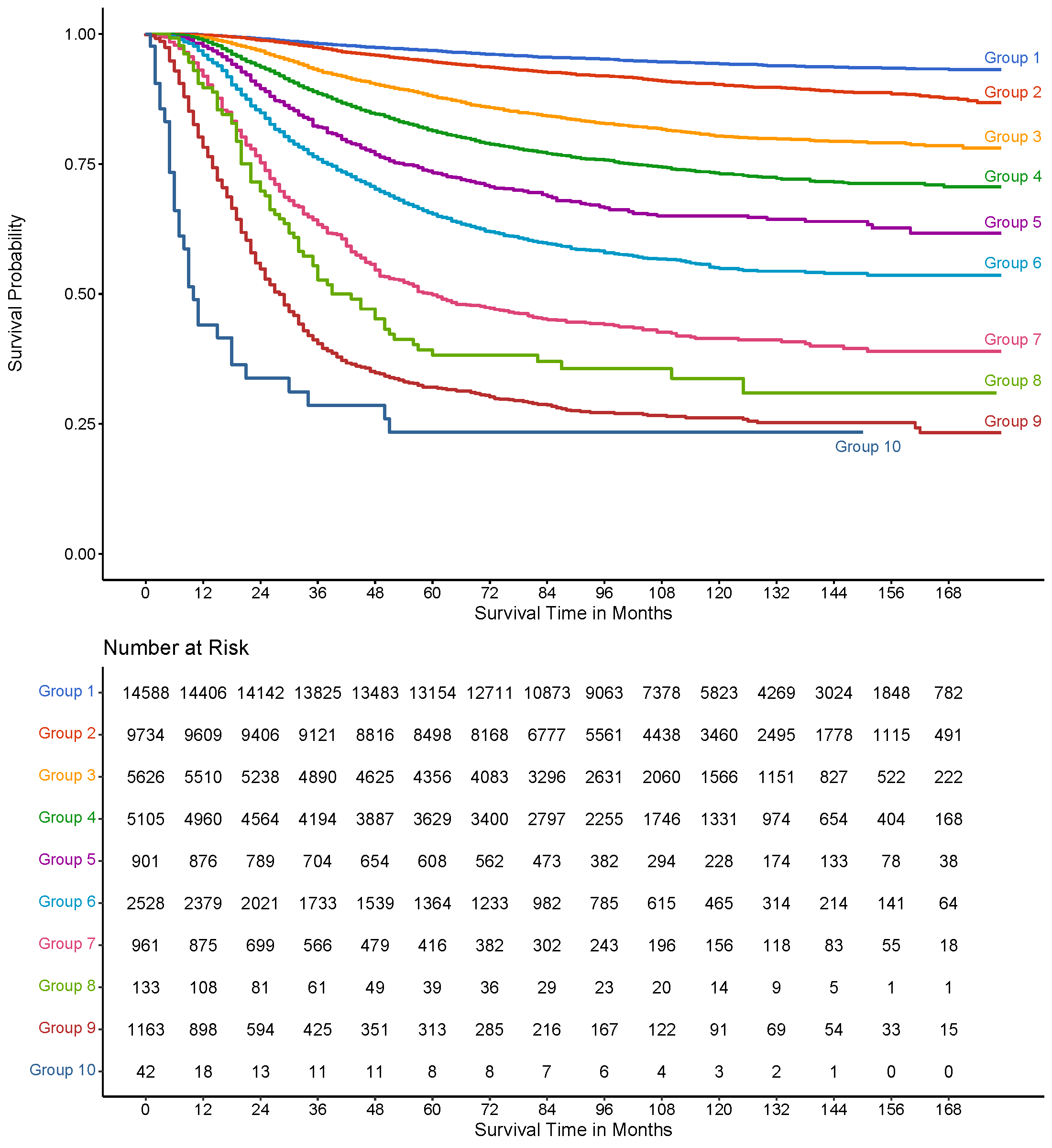

Supplement: S1 Fig — (TIF) [file pone.0257949.s002.tif]

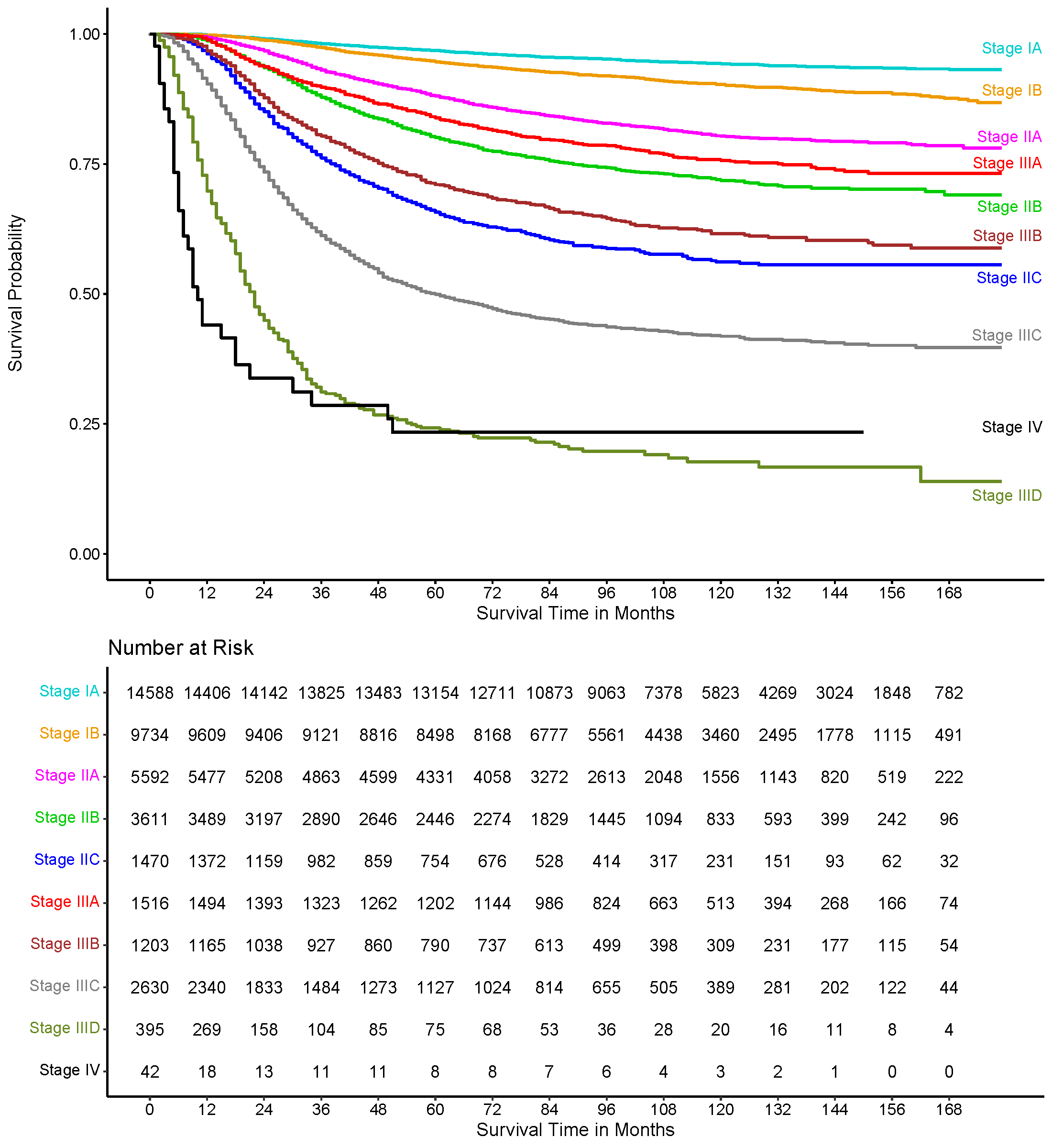

Supplement: S2 Fig — (TIF) [file pone.0257949.s003.tif]

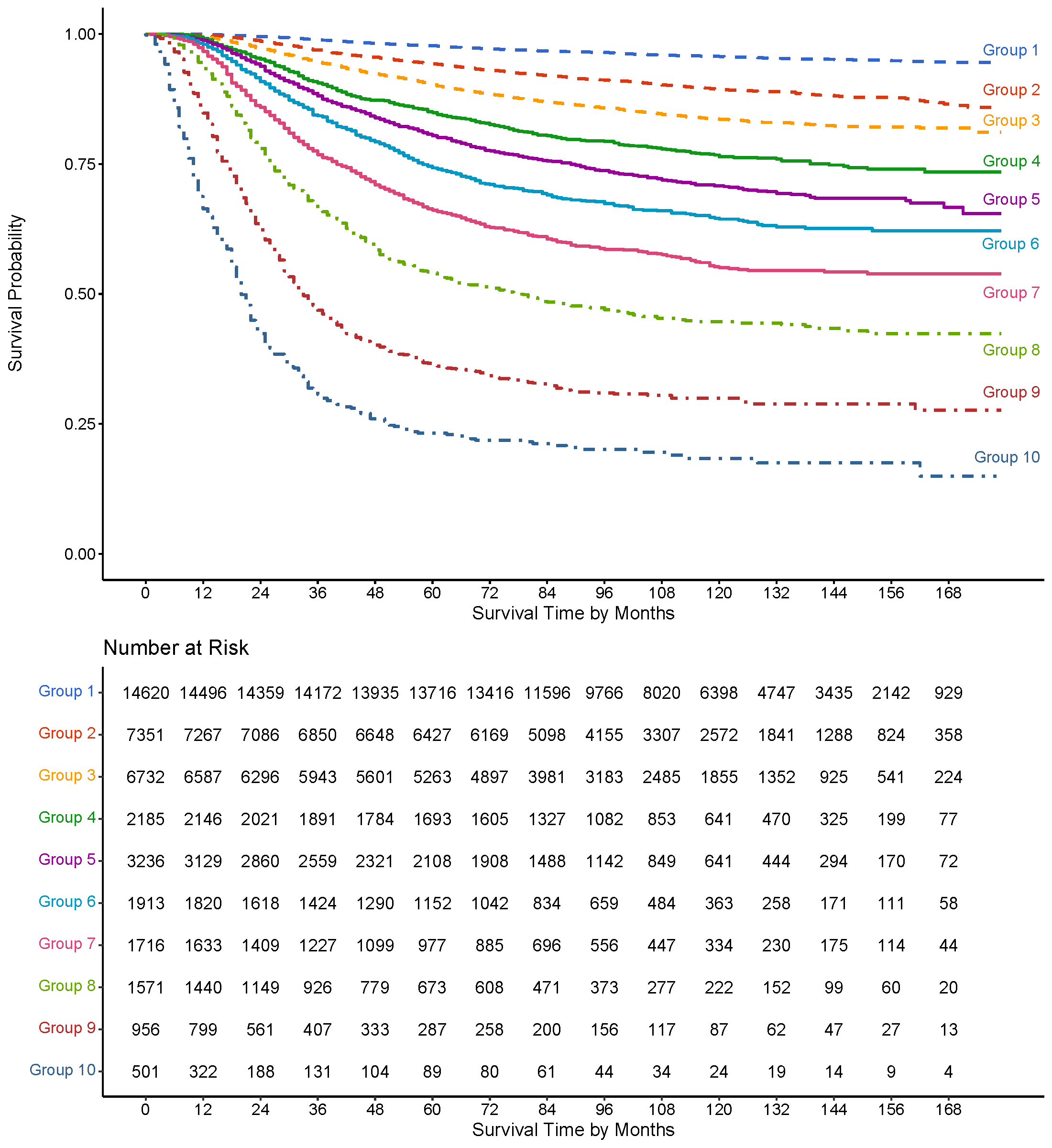

Supplement: S3 Fig — (TIF) [file pone.0257949.s004.tif]

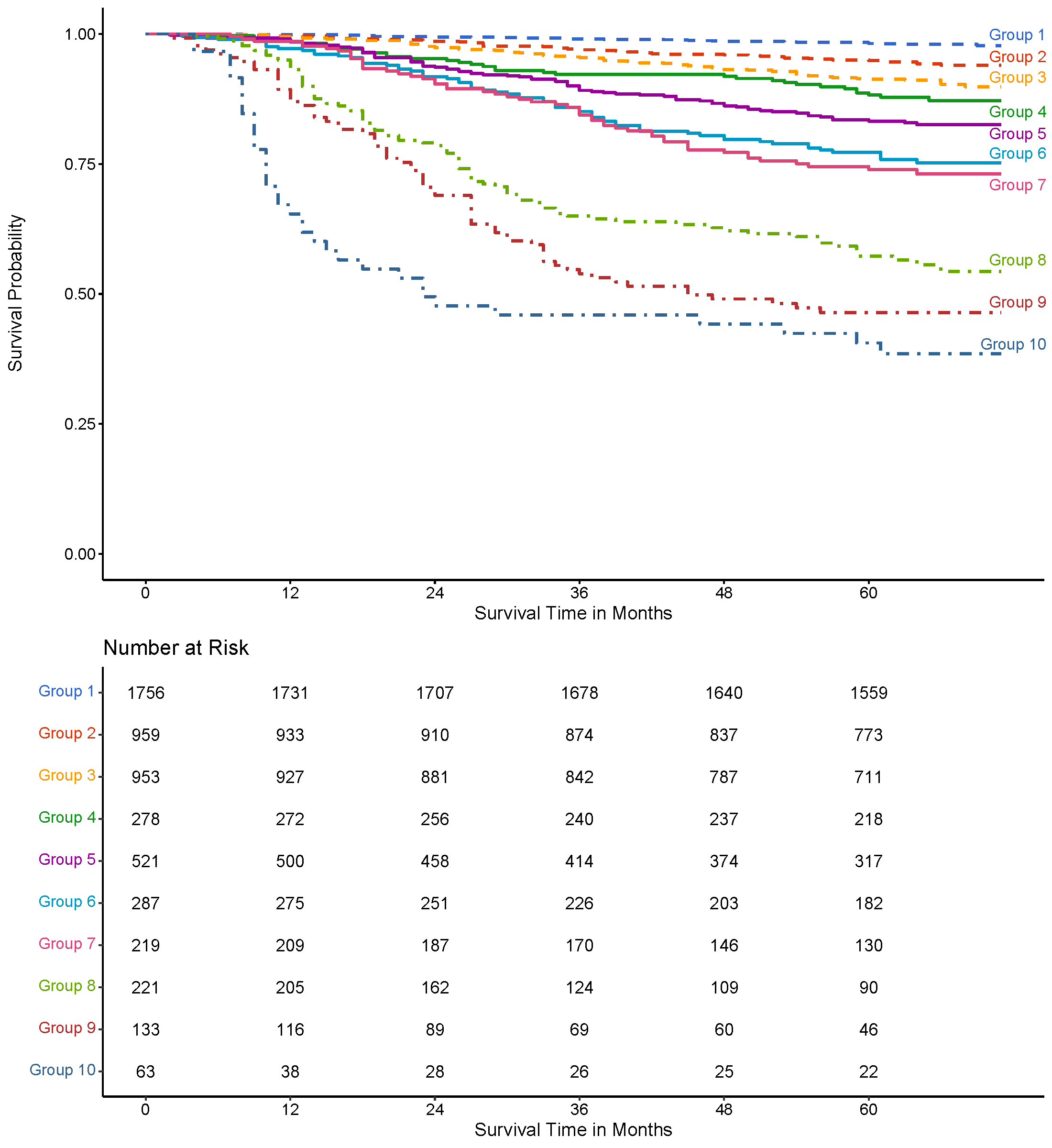

Supplement: S4 Fig — (TIF) [file pone.0257949.s005.tif]
