# Supplementary material for: Integrating additional factors into the TNM staging for cutaneous melanoma by machine learning
Source: PLoS One. 2021 Sep 30;16(9):e0257949. doi: 10.1371/journal.pone.0257949 (PMC8483349; doi:10.1371/journal.pone.0257949)
Supplement: S1 Table — Refer to AJCC Cancer Staging Manual [1] and SEER Research Data Record Description [2] for specifics of the 3rd column. (DOCX) [file pone.0257949.s006.docx]

**S1 Table. Definitions of levels of T, N, M, A, and S for SEER melanoma of the skin.**

| ***Prognostic Factors*** | ***Levels*** | ***Definitions*** | |
| --- | --- | --- | --- |
| *Primary Tumor (T)* |  | *Thickness* | *Ulceration status* |
|  | *T0: no evidence of primary tumor (e.g., unknown primary*  *or completely regressed melanoma)* | *Not applicable* | *Not applicable* |
|  | *T1a* | *<0.8mm* | *Without ulceration* |
|  | *T1b* | *<0.8 mm*  *0.8-1.0mm* | *With ulceration* |
|  | *T2a* | *>1.0-2.0 mm* | *Without ulceration* |
|  | *T2b* | *>1.0-2.0 mm* | *With ulceration* |
|  | *T3a* | *>2.0-4.0 mm* | *Without ulceration* |
|  | *T3b* | *>2.0-4.0 mm* | *With ulceration* |
|  | *T4a* | *>4.0mm* | *Without ulceration* |
|  | *T4b* | *>4.0mm* | *With ulceration* |
| *Regional Lymph Nodes (N)* |  | *Number of tumor-involved regional lymph node* | *Presence of in-transit,*  *satellite, and/or*  *microsatellite metastases* |
|  | *N0* | *No regional metastases*  *detected* | *No* |
|  | *N1a* | *One clinically occult (i.e., detected by SLN biopsy)* | *No* |
|  | *N1b* | *One clinically detected* | *No* |
|  | *N2a* | *Two or three clinically occult (i.e., detected by SLN biopsy)* | *No* |
|  | *N2b* | *Two or three, at least one of which was clinically detected* | *No* |
|  | *N2c* | *One clinically occult or clinically detected* | *Yes* |
|  | *N3* | *Four or more tumor-involved nodes or in-transit, satellite, and/or microsatellite metastases with two or more tumor-involved nodes, or any number of matted nodes without or with in transit, satellite, and/or microsatellite metastases* | |
| *Distant Metastasis (M)* | *M0* | *No evidence of distant metastasis* | |
|  | *M1* | *Evidence of distant metastasis* | |
| *Age (A)* | *A0* | *Age < 70* | |
|  | *A1* | *Age ≥ 70* | |
| *Sex (S)* | *S1* | *Male* | |
|  | *S2* | *Female* | |

Refer to AJCC Cancer Staging Manual [1] and SEER Research Data Record Description [2] for specifics of the 3rd column.

1. Amin MB, Edge S, Greene F, Byrd DR, Brookland RK, Washington, MK, et al. AJCC Cancer Staging Manual, 8th ed. Springer International Publishing. 2017.

1. SEER Research Data Record Description. Available online at: https://seer.cancer.gov/data-software/documentation/seerstat/nov2020/TextData.FileDescription.pdf (accessed 20 June 2021).
