# Supplementary material for: Integrating additional factors into the TNM staging for cutaneous melanoma by machine learning
Source: PLoS One. 2021 Sep 30;16(9):e0257949. doi: 10.1371/journal.pone.0257949 (PMC8483349; doi:10.1371/journal.pone.0257949)
Supplement: S2 Table — (DOCX) [file pone.0257949.s007.docx]

**S2 Table.** **EACCD and AJCC grouping of melanoma of the skin patients according to T, N, and M.**

| ***T*** | ***N*** | ***M*** | ***EACCD prognostic***  ***group*** | ***8th AJCC***  ***Staging Manual***  ***stage*** |
| --- | --- | --- | --- | --- |
| T1a | N0 | M0 | 1 | IA |
| T1b | N0 | M0 | 1 | IA |
| T2a | N0 | M0 | 2 | IB |
| T1a | N2a | M0 | 3 | IIIA |
| T2b | N0 | M0 | 3 | IIA |
| T3a | N0 | M0 | 3 | IIA |
| T1a | N1a | M0 | 4 | IIIA |
| T1b | N1a | M0 | 4 | IIIA |
| T2a | N1a | M0 | 4 | IIIA |
| T2a | N2a | M0 | 4 | IIIA |
| T2a | N2c | M0 | 4 | IIIC |
| T3b | N0 | M0 | 4 | IIB |
| T4a | N0 | M0 | 4 | IIB |
| T0 | N1b | M0 | 5 | IIIB |
| T0 | N3 | M0 | 5 | IIIC |
| T2a | N1b | M0 | 5 | IIIB |
| T2b | N1a | M0 | 5 | IIIB |
| T3a | N1a | M0 | 5 | IIIB |
| T1b | N2a | M0 | 6 | IIIA |
| T2a | N2b | M0 | 6 | IIIB |
| T3a | N1b | M0 | 6 | IIIB |
| T3a | N2a | M0 | 6 | IIIB |
| T3a | N2b | M0 | 6 | IIIB |
| T3b | N1a | M0 | 6 | IIIC |
| T4a | N1a | M0 | 6 | IIIC |
| T4a | N1b | M0 | 6 | IIIC |
| T4b | N0 | M0 | 6 | IIC |
| T1b | N1b | M0 | 7 | IIIB |
| T2a | N3 | M0 | 7 | IIIC |
| T2b | N2a | M0 | 7 | IIIB |
| T3a | N3 | M0 | 7 | IIIC |
| T3b | N1b | M0 | 7 | IIIC |
| T3b | N2a | M0 | 7 | IIIC |
| T3b | N2b | M0 | 7 | IIIC |
| T4a | N2a | M0 | 7 | IIIC |
| T4b | N1a | M0 | 7 | IIIC |
| T4a | N2b | M0 | 8 | IIIC |
| T4b | N2c | M0 | 8 | IIIC |
| T1b | N3 | M0 | 9 | IIIC |
| T2b | N3 | M0 | 9 | IIIC |
| T3b | N3 | M0 | 9 | IIIC |
| T4a | N3 | M0 | 9 | IIIC |
| T4b | N1b | M0 | 9 | IIIC |
| T4b | N2a | M0 | 9 | IIIC |
| T4b | N2b | M0 | 9 | IIIC |
| T4b | N3 | M0 | 9 | IIID |
| T4b | N3 | M1 | 10 | IV |
