# Supplementary material for: Integrating additional factors into the TNM staging for cutaneous melanoma by machine learning
Source: PLoS One. 2021 Sep 30;16(9):e0257949. doi: 10.1371/journal.pone.0257949 (PMC8483349; doi:10.1371/journal.pone.0257949)
Supplement: S3 Table — (DOCX) [file pone.0257949.s008.docx]

**S3 Table. EACCD grouping of melanoma of the skin patients according to T, N, M, A, and S.**

| ***T*** | ***N*** | ***M*** | ***A*** | ***S*** |
| --- | --- | --- | --- | --- |
| Group 1 | | | | |
| T1a | N0 | M0 | A0 | S1 |
| T1a | N0 | M0 | A0 | S2 |
| T1b | N0 | M0 | A0 | S1 |
| T1b | N0 | M0 | A0 | S2 |
| T2a | N0 | M0 | A0 | S2 |
| Group 2 | | | | |
| T1a | N0 | M0 | A1 | S2 |
| T1b | N0 | M0 | A1 | S2 |
| T2a | N0 | M0 | A0 | S1 |
| T2a | N0 | M0 | A1 | S2 |
| T2b | N0 | M0 | A0 | S2 |
| T3a | N0 | M0 | A0 | S2 |
| Group 3 | | | | |
| T1a | N0 | M0 | A1 | S1 |
| T1a | N1a | M0 | A0 | S1 |
| T1b | N0 | M0 | A1 | S1 |
| T1b | N1a | M0 | A0 | S2 |
| T2a | N0 | M0 | A1 | S1 |
| T2a | N1a | M0 | A0 | S2 |
| T2b | N0 | M0 | A0 | S1 |
| T3a | N0 | M0 | A0 | S1 |
| T4a | N0 | M0 | A0 | S2 |
| Group 4 | | | | |
| T1a | N1a | M0 | A0 | S2 |
| T1a | N2a | M0 | A0 | S1 |
| T1b | N1a | M0 | A0 | S1 |
| T2a | N1a | M0 | A0 | S1 |
| T2a | N2a | M0 | A0 | S2 |
| T2a | N2c | M0 | A0 | S1 |
| T2b | N1a | M0 | A0 | S2 |
| T3a | N0 | M0 | A1 | S2 |
| T3b | N0 | M0 | A0 | S2 |
| T4a | N0 | M0 | A0 | S1 |
| Group 5 | | | | |
| T2a | N2a | M0 | A0 | S1 |
| T2a | N2b | M0 | A0 | S1 |
| T2a | N2c | M0 | A1 | S1 |
| T2b | N0 | M0 | A1 | S1 |
| T2b | N0 | M0 | A1 | S2 |
| T3a | N0 | M0 | A1 | S1 |
| T3a | N1a | M0 | A0 | S2 |
| T3a | N1a | M0 | A1 | S2 |
| T3b | N0 | M0 | A0 | S1 |
| T3b | N0 | M0 | A1 | S2 |
| T4a | N0 | M0 | A1 | S2 |
| Group 6 | | | | |
| T0 | N1b | M0 | A0 | S1 |
| T0 | N3 | M0 | A0 | S1 |
| T2a | N1b | M0 | A0 | S1 |
| T3a | N1a | M0 | A0 | S1 |
| T3a | N1b | M0 | A0 | S1 |
| T3b | N0 | M0 | A1 | S1 |
| T3b | N1a | M0 | A0 | S2 |
| T4a | N0 | M0 | A1 | S1 |
| T4a | N1a | M0 | A0 | S2 |
| T4b | N0 | M0 | A0 | S2 |
| Group 7 | | | | |
| T1a | N1a | M0 | A1 | S1 |
| T1b | N1a | M0 | A1 | S1 |
| T1b | N2a | M0 | A0 | S1 |
| T2a | N1a | M0 | A1 | S1 |
| T2a | N1a | M0 | A1 | S2 |
| T2b | N1a | M0 | A0 | S1 |
| T2b | N1a | M0 | A1 | S1 |
| T3a | N2a | M0 | A0 | S1 |
| T3a | N2a | M0 | A0 | S2 |
| T3a | N2b | M0 | A0 | S1 |
| T3b | N1a | M0 | A0 | S1 |
| T3b | N2a | M0 | A0 | S2 |
| T4a | N1a | M0 | A0 | S1 |
| T4a | N1b | M0 | A0 | S1 |
| T4b | N0 | M0 | A0 | S1 |
| T4b | N0 | M0 | A1 | S2 |
| Group 8 | | | | |
| T1b | N1b | M0 | A0 | S1 |
| T2a | N3 | M0 | A0 | S1 |
| T2b | N2a | M0 | A0 | S1 |
| T3a | N1a | M0 | A1 | S1 |
| T3a | N2a | M0 | A1 | S1 |
| T3a | N3 | M0 | A0 | S1 |
| T3a | N3 | M0 | A0 | S2 |
| T3b | N1a | M0 | A1 | S1 |
| T3b | N1a | M0 | A1 | S2 |
| T3b | N1b | M0 | A0 | S1 |
| T3b | N2a | M0 | A0 | S1 |
| T3b | N2b | M0 | A0 | S1 |
| T4a | N1a | M0 | A1 | S1 |
| T4a | N2a | M0 | A0 | S1 |
| T4b | N0 | M0 | A1 | S1 |
| T4b | N1a | M0 | A0 | S1 |
| T4b | N1a | M0 | A0 | S2 |
| T4b | N2b | M0 | A0 | S2 |
| T4b | N2c | M0 | A0 | S1 |
| T4b | N2c | M0 | A1 | S2 |
| Group 9 | | | | |
| T1b | N3 | M0 | A0 | S1 |
| T2b | N3 | M0 | A0 | S1 |
| T3b | N2a | M0 | A1 | S1 |
| T3b | N3 | M0 | A0 | S1 |
| T3b | N3 | M0 | A0 | S2 |
| T4a | N1a | M0 | A1 | S2 |
| T4a | N2b | M0 | A0 | S1 |
| T4a | N3 | M0 | A0 | S1 |
| T4b | N1a | M0 | A1 | S1 |
| T4b | N1a | M0 | A1 | S2 |
| T4b | N1b | M0 | A0 | S1 |
| T4b | N1b | M0 | A1 | S1 |
| T4b | N2a | M0 | A0 | S1 |
| T4b | N2a | M0 | A0 | S2 |
| T4b | N2a | M0 | A1 | S1 |
| T4b | N2b | M0 | A0 | S1 |
| T4b | N2c | M0 | A1 | S1 |
| T4b | N3 | M0 | A0 | S2 |
| Group 10 | | | | |
| T3b | N3 | M0 | A1 | S1 |
| T4a | N3 | M0 | A0 | S2 |
| T4a | N3 | M0 | A1 | S1 |
| T4b | N2b | M0 | A1 | S1 |
| T4b | N3 | M0 | A0 | S1 |
| T4b | N3 | M0 | A1 | S1 |
| T4b | N3 | M0 | A1 | S2 |
| T4b | N3 | M1 | A0 | S1 |
